# Mandate or Not Mandate: Knowledge, Attitudes, and Practices of Italian Occupational Physicians towards SARS-CoV-2 Immunization at the Beginning of Vaccination Campaign

**DOI:** 10.3390/vaccines9080889

**Published:** 2021-08-11

**Authors:** Matteo Riccò, Pietro Ferraro, Simona Peruzzi, Federica Balzarini, Silvia Ranzieri

**Affiliations:** 1 Servizio di Prevenzione e Sicurezza Negli Ambienti di Lavoro (SPSAL), AUSL-IRCCS di Reggio Emilia, I-42122 Reggio Emilia, RE, Italy; 2 Hospital S Camillo De Lellis, Occupational Health and Safety Service, ASL Foggia, I-41121 Foggia, FG, Italy; dott.pietro.ferraro@gmail.com; 3 Laboratorio Analisi Chimico Cliniche e Microbiologiche, Ospedale Civile di Guastalla, AUSL-IRCCS di Reggio Emilia, I-42016 Guastalla, RE, Italy; simona.peruzzi@ausl.re.it; 4 Dipartimento P.A.A.P.S.S., Servizio Autorizzazione e Accreditamento, Agenzia di Tutela della Salute (ATS) di Bergamo, I-24121 Bergamo, BG, Italy; federica.balzarini@ats-bg.it; 5 Department of Medicine and Surgery, University of Parma, I-43126 Parma, PR, Italy; silvia.ranzieri@unipr.it

**Keywords:** knowledge, occupational physicians, risk perception, SARS-CoV-2, COVID-19

## Abstract

Vaccinations used to prevent coronavirus disease (COVID-19)—the disease caused by severe acute respiratory syndrome coronavirus 2 (SARS-CoV-2)—are critical in order to contain the ongoing pandemic. However, SARS-CoV-2/COVID-19 vaccination rates have only slowly increased since the beginning of the vaccination campaign, even with at-risk workers (e.g., HCWs), presumptively because of vaccine hesitancy. Vaccination mandates are considered instrumental in order to rapidly improve immunization rates (but they minimize the impact of vaccination campaigns). In this study, we investigated the acceptance (i.e., knowledge, attitudes, and practices) from occupational physicians (OPs)) in regard to SARS-CoV-2/COVID-19 vaccination mandates. A total of 166 OPs participated in an internet-based survey by completing structured questionnaires. Adequate, general knowledge of SARS-CoV-2/COVID-19 was found in the majority of OPs. High perception of SARS-CoV-2 risk was found in around 80% of participants (79.5% regarding its occurrence, 81.9% regarding its potential severity). SARS-CoV-2/COVID-19 vaccination was endorsed by 90.4% of respondents, acceptance for SARS-CoV-2 vaccine was quite larger for mRNA formulates (89.8%) over adenoviral ones (59.8%). Endorsement of vaccination mandates was reported by 60.2% of respondents, and was more likely endorsed by OPs who exhibited higher concern for SARS-CoV-2 infection occurrence (odds ratio 3.462, 95% confidence intervals 1.060–11.310), who were likely to accept some sort of payment/copayment for SARS-CoV-2/COVID-19 vaccination (3.896; 1.607; 9.449), or who were more likely to believe HCWs not vaccinates against SARS-CoV-2 as unfit for work (4.562; 1.935; 10.753). In conclusion, OPs exhibited wide acceptance of SARS-CoV-2/COVID-19 vaccinations, and the majority endorsed vaccination mandates for HCWs, which may help improve vaccination rates in occupational settings.

## 1. Introduction

On 11 March, 2020, the World Health Organization declared coronavirus disease 2019 (COVID-19), the disease caused by severe acute respiratory syndrome coronavirus 2 (SARS-CoV-2), a pandemic [1]. Since then, as of August 2021, there were more than 204 million cases and approximately 4 million deaths due to SARS-CoV-2, and even more disruptions impacting societies and economies worldwide [2]. Therefore, development and deployment of vaccines that are able to stop the COVID-19 pandemic have become a global priority [3].

In December 2020, the U.S. Food and Drug Administration (FDA) and European Medicines Agency (EMA) issued emergency use authorization for two messenger RNA (mRNA) vaccines, based on data reportedly demonstrating 95% efficacy. This led to vaccination campaigns that primarily prioritized healthcare workers (HCWs) [4,5,6,7]. Even though SARS-CoV-2 vaccinations are shown to be effective against COVID-19, which is beneficial for HCWs and their contacts (both household and professional) [8], there are still vaccine-hesitant HCWs [9]. Moreover, the option of issuing mandatory COVID-19 vaccinations for high risk workers is being debated [8,10].

In this regard, where legal frameworks take place, occupational physicians (OPs) may become key players who encourage SARS-CoV-2 vaccine acceptance [3,9,11]. OPs are the medical professionals responsible for health promotion in workplaces; they directly contribute to immunization programs by applying and tailoring official recommendations [12,13]. Moreover, OPs are diffusely involved in risk communication, essentially disseminating information to HCWs about health risks. For instance, the Italian Occupational Health and Safety Legislation requires OPs to inquire about vaccination history, to recall vaccination status, and to inform workers about the pros and cons of the recommended vaccinations [13,14]. Unfortunately, previous studies show that OPs may report significant misunderstandings in regard to vaccinations and vaccination policies [13,14,15,16,17]. In other words, OPs not only often fail to overcome the gaps found between public health professionals and vaccine objectors, but, being themselves possibly affected by some degree of vaccine hesitancy, they might also impair the shared efforts that improve vaccination rates in occupational groups and in the general population, with obvious detrimental effects (i.e., when dealing with the ongoing SARS-CoV-2 pandemic).

Therefore, the main goal of this study was to characterize the acceptance of SARS-CoV-2 vaccines among Italian OPs via their respective knowledge (i.e., awareness of official recommendations), attitudes (i.e., propensity towards vaccinations), and practices (i.e., actual uptake of vaccinations), or KAP. Moreover, we assessed their willingness to pay for receiving a SARS-CoV-2 vaccine, the attitudes of sampled OPs towards issuing mandatory SARS-CoV-2 vaccines in high-risk groups, and respective explanatory factors. Eventually, our results may be useful for targeting specific informative and educative campaigns dedicated to OPs, which could, in turn, improve the general acceptance of SARS-CoV-2 vaccinations.

## 2. Materials and Methods

### 2.1. Study Design

A cross-sectional questionnaire-based study was performed between 1 January 2021 and 13 January 2021, involving OPs participating in seven different private Facebook groups and four closed forums, focusing on occupational medicine (where application was officially limited to OPs. The group pages had approximately 2034 unique members (i.e., users of a membership-based organization that has a distinctive profile), but no information could be obtained regarding cross-inscriptions in-between the groups, nor how many members were active Facebook users at the time of the survey. The mode of an online survey was chosen due to the difficulty in conducting a face-to-face study amid the ongoing COVID-19 outbreak.

To share the study invitation—the chief researcher contacted the administrators, requesting preventive authorization to post the questionnaire link, including a short description of the aims of the survey. Facebook users who clicked on the link were provided with the full study information, an opportunity to give their informed consent, and a web link to the survey (Google Forms; Google LLC; Menlo Park, CA, USA). The survey was conducted in Italian. To be included in the sample, participants had to live and work in Italy as OPs. If a potential participant did not match the inclusion criteria, the survey closed. Similarly, participants were asked whether they assisted any healthcare providers. OPs who were not involved in healthcare settings were excluded from the study. The survey was anonymous, and no personal data, such as name, IP address, email address, or personal information unnecessary to the survey, was requested, saved, or tracked. No monetary or other compensation was offered to the participants.

### 2.2. Questionnaire

The questionnaire was formulated in Italian, and its test–retest reliability was preventively assessed through a survey on 15 OPs completing the questionnaire at two different points in time. The beta-testing questionnaires were ultimately excluded from the final analyses. All questions were self-reported, and not externally validated. According to the health belief model [12,18], we assumed that KAP and the eventual acceptance of SARS-CoV-2 vaccination depended on: (a) availability, i.e., the actual existence of an effective vaccine; (b) access to the vaccine; (c) perceived health risk, which depends on the trade-off between the vaccine (i.e., occurrence and severity of potential side effects) and COVID-19 (i.e., its prevalence and severity); (d) information on benefits, risks, and access pathways; (e) previous experience with other vaccines and exposure to diseases, as this affects risk perception; and (f) sociodemographic factors, including age, education level, gender, and more. Therefore, the final questionnaire included the following sections.

#### 2.2.1. Individual Characteristics

Age (arbitrarily dichotomized as <50-year-old vs. ≥50-year-old), sex, seniority as physician (arbitrarily dichotomized as <15 years vs. ≥15 years), whether they or any of their relatives had received a diagnosis of COVID-19 (yes vs. no), if they worked as OPs in healthcare settings affiliated with the National Health Service, and the Italian Region where the professional mainly worked and lived. The latter factor was eventually dichotomized as Northern Italy (i.e., Valle d’Aosta, Piemonte, Lombardia, Liguria, Emilia Romagna, Veneto, Friuli Venezia Giulia, Autonomous Provinces of Trento and Bolzano) vs. all other regions.

#### 2.2.2. Knowledge Test

Participants received a knowledge test containing a set of 16 true–false statements, elaborated through an extensive literature review, covering typical misconceptions about COVID-19 and SARS-CoV-2 infections (e.g., “Main complications of COVID-19 is represented by respiratory distress syndrome”; TRUE) that was originally validated in a larger scale survey on HCWs [19]. A general knowledge score (GKS) was then calculated as the sum of correctly and incorrectly marked recommendations. When the participants answered correctly, +1 was added to the sum score, whereas a wrong indication or a missing/”don’t know” answer added 0 to the sum score. GKS was then dichotomized by median value in higher vs. lower knowledge status.

#### 2.2.3. Risk Perception

Participants were initially asked whether or not they perceived healthcare settings as high risks for SARS-CoV-2 infection, and whether they, as OPs, felt they were at an increased risk for COVID-19, when compared to the general population. The items were presented as a 5-point Likert scale (i.e., from 1 “totally disagree”, to 5 “totally agree”) and eventually dichotomized as somewhat agreeing (i.e., agree to totally agree) vs. somewhat disagreeing (i.e., totally disagree to neutral).

Then, participants were asked to rate the perceived severity (C^INF^) and the perceived frequency (I^INF^) of SARS-CoV-2 infections in the general population by means of a fully labeled 5-point Likert scale. The available options ranged from “not significant” (i.e., “of no significant concern”, score 1) to “very significant” (i.e., “of very high concern”, score 5). Similarly, they were asked to rate the perceived severity (C^VAC^) and frequency (I^VAC^) of side effects associated with SARS-CoV-2 mRNA-based vaccines, as the only ones available at the time of the study. As perceived risk was defined as a function of the perceived probability of an event and its expected consequences [12,20], the corresponding risk perception score (RPS) was separately calculated for vaccines and SARS-CoV-2 infection as follows:

RPS = I × C(1)

Eventual RPS for COVID-19 and mRNA vaccines were then dichotomized by median value in high (i.e., >median) vs. low risk perception (i.e., ≤median).

#### 2.2.4. Attitudes and Practices

We asked participants about their trust in vaccines, i.e., were they instrumental in preventing infectious diseases, in general, and then we focused on seasonal influenza vaccines and COVID-19 vaccines (i.e., mRNA-based vaccines, adenovirus-based vaccines, any). Similarly, a series of perceived barriers (e.g., inappropriate vaccine safety, inappropriate vaccine efficacy, etc.) and facilitators (e.g., willingness to protect himself/herself; willingness to protect friends, relatives, etc.) were presented to the participants. All aforementioned items were presented as a 5-point fully-labeled Likert scale, ranging from “totally disagree” to “totally agree”, and were dichotomized as somewhat agreeing (i.e., agree to totally agree) vs. somewhat disagreeing (i.e., totally disagree to neutral).

Participants were then asked about their willingness to pay for a SARS-CoV-2 vaccine (i.e., should be provided for free; participation to the expenditure; up to EUR 10/dose; between EUR 10 and 49/dose; between EUR 50 and 99/dose; between EUR 100 and 199/dose; EUR 200 or more/dose); whether they believed that the SARS-CoV-2 vaccine should be mandatory or not, and whether HCWs who did not receive the SARS-CoV-2 vaccine should be considered unfit for work in high risk settings (i.e., permanently unfit; unfit but temporarily reassigned to other low-risk tasks; still fit for work).

### 2.3. Ethical Considerations

Before consenting to the survey, participants were informed that all information would be gathered anonymously and handled confidentially. Participation was voluntary, and the questionnaire was collected only from subjects who had expressed consent to study participation. As individual participants cannot be identified based on the presented material, this study caused no plausible harm or stigma to participating individuals. As the study had an anonymous, observational design, and did not include clinical data about patients, nor configured itself as a clinical trial, a preliminary evaluation by an Ethical Committee was not required, according to Italian law [21].

### 2.4. Data Analysis

Continuous variables were initially tested for normal distribution (D’Agostino and Pearson omnibus normality test), where the corresponding *p* value was <0.10, “normal” distribution was assumed as rejected and variables were compared through the Mann–Whitney or Kruskal–Wallis test for multiple independent samples. On the other hand, variables passing the normality check (D’Agostino and Pearson *p* value ≥ 0.10) were compared using the Student’s t test or ANOVA, where appropriate. Categorical variables were reported as percentage values, and their distribution, in respect to the outcome variable (promoting a mandatory status for SARS-CoV-2 vaccines) was initially analyzed through chi-squared test. All categorical variables that, at univariate analysis, were significantly associated with a positive attitude towards a mandatory status for SARS-CoV-2 vaccines (i.e., *p* < 0.05) were included in a stepwise binary logistic regression analysis model, in order to calculate the adjusted odds ratios (aOR) and their respective 95% confidence intervals (95% CI). All statistical analyses were performed by means of IBM SPSS Statistics 24.0 for Macintosh (IBM Corp. Armonk, NY, USA).

## 3. Results

### 3.1. Descriptive Analysis

As shown in Table 1, a total of 166 OPs (8.2% of the potentially eligible population) participated in the inquiry. Around 1/3 of all respondents were aged 50 years or older (36.1%; mean age 49.1 years ± 10.7), with a mean seniority of 22.2 years ± 10.3 (80.7% having a seniority ≥ 15 years); 59.6% were females and 40.4% males. As for inclusion criteria—all participants had experience as OPs of healthcare providers, but the majority (i.e., 59.0%) were involved in the health surveillance of HCWs in healthcare settings affiliated with the Italian National Health Service (provincial or even regional level hospitals).

Overall, 36.1% of participants were from northern regions (i.e., Valle d’Aosta, Piemonte, Liguria, Lombardia, Veneto, Trentino-Südtirol, Friuli-Venezia-Giulia, Emilia-Romagna), with 31.3% of respondents from Central Italy (i.e., Toscana, Umbria, Marche, Abruzzo, Lazio), and the residual 32.5% from southern regions (i.e., Campania, Molise, Puglia, Basilicata, Calabria) and major islands of Sicilia and Sardinia. Of them, 10.2% were previously diagnosed with SARS-CoV-2 infection, while 25.3% reported a diagnosis of COVID-19 among friends or relatives. The most commonly reported information sources on SARS-CoV-2/COVID-19 were websites from international and governmental agencies (88.0%), followed by formation courses (70.5%), while conventional media (21.7%), new media (16.9%), friends/relatives (4.8%), and even colleagues (1.8%) were reported as residual sources.

### 3.2. Assessment of Knowledge about SARS-CoV-2

**Figure 1:**
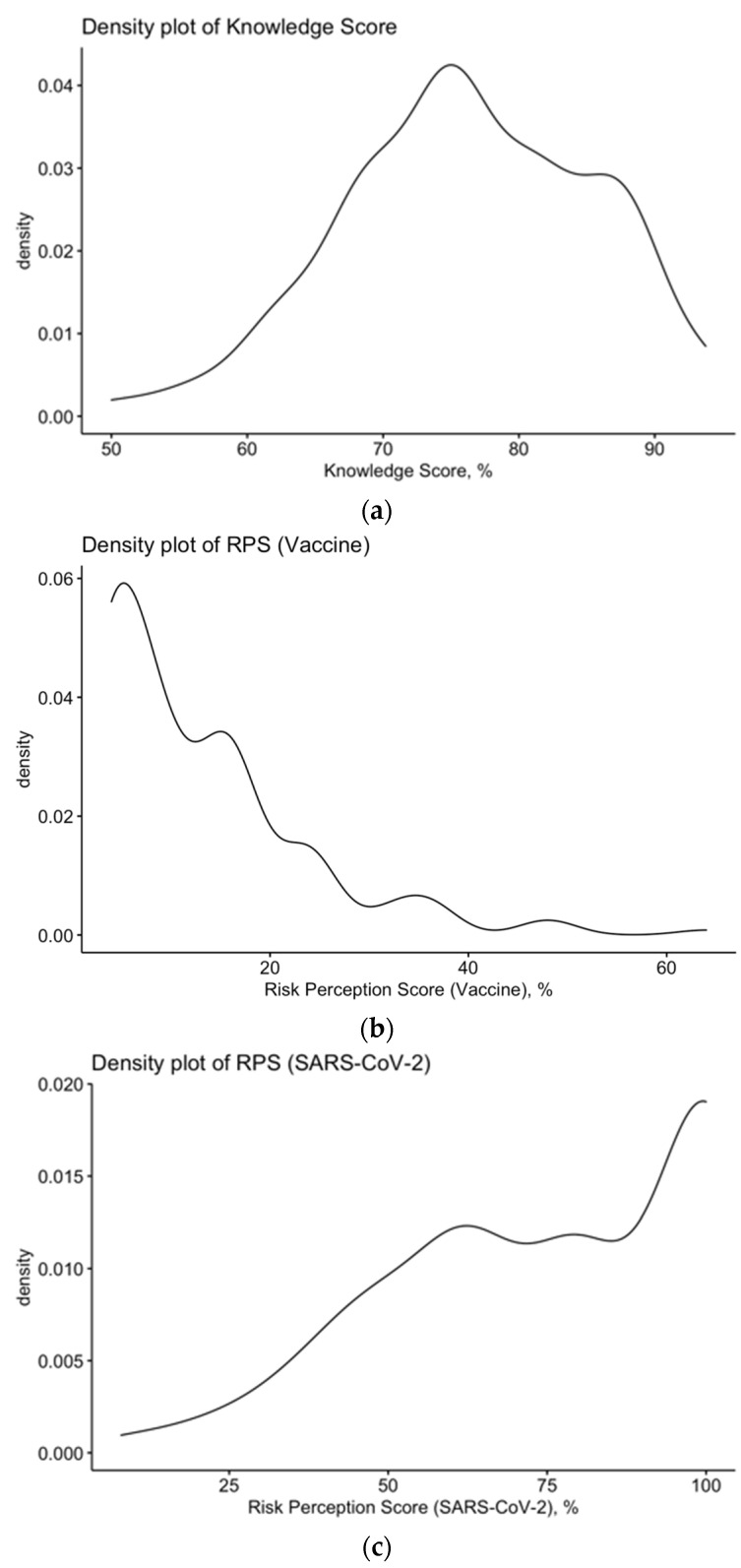
Density plot for (left to right): (**a**) knowledge score (%); (**b**) risk perception score (%) for mRNA vaccine; (**c**) risk perception score (%) for SARS-CoV-2. All cumulative scores appeared not-normally distributed (D’Agostino–Pearson *p* value < 0.001 for all scores).

The internal consistency coefficient amounted to Cronbach’s alpha = 0.717. The overall understanding of COVID-19 among participants was skewed (D’Agostino–Pearson *p* value < 0.001; Figure 1), but quite good, as, after normalization, the mean GKS was generally high (76.3% ± 9.3; actual range 50.0%–93.8%; median 75.0%).

However, as shown in Table 2, only 39.8% of respondents were aware that, at the time of the survey, the case fatality ratio for COVID-19 in Italy was approximately 1.0%. Similarly, less than half of the sampled OPs (44.0%) were aware that, by January 2021, no adenovirus-based SARS-CoV-2 vaccine had been approved by the EMA, and only 56.6% of participants recalled the official recommendations for delaying SARS-CoV-2 immunization with mRNA-based preparations in pregnant women.

### 3.3. Assessment of Attitudes and Practices

As shown in Table 3, the majority of respondents (95.8%) reported high or very high trust in vaccines as instrumental in preventing infectious diseases, but only 63.3% reported being vaccinated against seasonal influenza vaccine during at least 4 of the 5 winter seasons. Focusing on COVID-19 vaccines—90.4% of respondents acknowledged a high or even very high acceptance, which peaked to 89.8% for mRNA-based preparations compared to 51.2% for adenovirus-based vaccines.

Interestingly, the majority of participants indicated they would participate in the expenditure for the SARS-CoV-2 vaccine. While 29.5% did not indicate a range of expenditure, 9.6% accepted an out-of-pocket expenditure, ranging from EUR 50 to 99, and again from EUR 100 to 199, while 9.0% accepted an expenditure of EUR 200 or more, 5.4% up to EUR 10, and 10 to EUR 49. On the contrary, around a third of respondents (31.3%) recommended that the vaccine had to be provided for free.

The majority of respondents (60.2%) were somewhat favorable toward mandatory status of the SARS-CoV-2 vaccine, either with (27.1%) and without (33.1%) fines for the vaccine-hesitant, while only 21.7% recommended a voluntary basis, and 17.5% were favorable to the official recommendation in high-risk subjects and/or workers, including HCWs. On the contrary, only 1 participant (0.6%) was formally against the SARS-CoV-2 vaccine, because of an alleged unsafe profile. Not coincidentally, the large majority of participants (69.9%) would consider vaccine-hesitant HCWs as unfit for work in healthcare settings, either permanently (19.9%) or temporarily (50.0%), with transitory reassignment to low-risk tasks. In summary, the internal consistency coefficient for reported attitudes amounted to Cronbach’s alpha = 0.766 (i.e., the questionnaire was characterized by acceptable reliability).

### 3.4. Assessment of the Risk Perception

As total of 136 out of 166 respondents (81.9%) acknowledged COVID-19 as a severe disease, with a similar share of participants reporting the infection as commonly reported (79.5%), with an eventual RPS of 74.5% ± 24.3. Moreover, 62.7% of respondents reported a perceived high risk for COVID-19 infection among OPs. On the contrary, side effects of SARS-CoV-2 mRNA-based vaccines were acknowledged as severe, and frequently reported by around 5% of participants (5.4% and 4.2%, respectively), with a resulting RPS of 13.0% ± 10.6.

### 3.5. Perceived Facilitators and Barriers

In regard to the perceived facilitators towards SARS-CoV-2 vaccinations in the workplaces (Table 4), the majority of respondents acknowledged a willingness to protect friends/relatives (66.3%), followed by the perception of being among a high risk-group (48.8%), the aim of protecting himself/herself (47.0%), the acknowledgement of COVID-19 as a severe disease (43.4%), and the willingness to avoid COVID-19 (36.1%), and its complications (34.3%), while only 2.4% reported a lack of confidence in alternative treatments. Moreover, we inquired about a series of factors more specifically involved in healthcare settings; among them, the most frequently reported was the lack of confidence in PPE (91.6%), followed by the inappropriate risk perception in most HCWs (85.5%), the limited reliability of non-pharmaceutical interventions in healthcare settings (68.1%), and the scarce reliability of tracing and tracking of SARS-CoV-2 cases among HCWs.

Regarding the possible barriers—approximately half of respondents identified doubts in vaccine safety (47.0%), followed by the difficult supplying of vaccines (24.1%), doubts on vaccine efficacy (19.9%), and lack of confidence in the pharmaceutical industry (19.3%). Interestingly, doubts on vaccine efficacy were associated with lower vaccine acceptance (*p* = 0.029). Doubts on the effective severity of COVID-19 and on the actual risk status of the workers were reported by only 18.1% and 12.0% of participants. Eventually, lack of confidence in NHS (10.2%) and its personnel (4.2%), as well as higher confidence in alternative approaches, such as hyperimmune plasma (5.4%), and hydroxychloroquine (4.8%), were somewhat residual among study participants. The internal consistency coefficient for perceived facilitators and barriers amounted to Cronbach’s alpha = 0.741 and 704, respectively, suggesting the acceptable reliability of the questionnaire’s corresponding items.

### 3.6. Univariate Analysis

**Figure 2:**
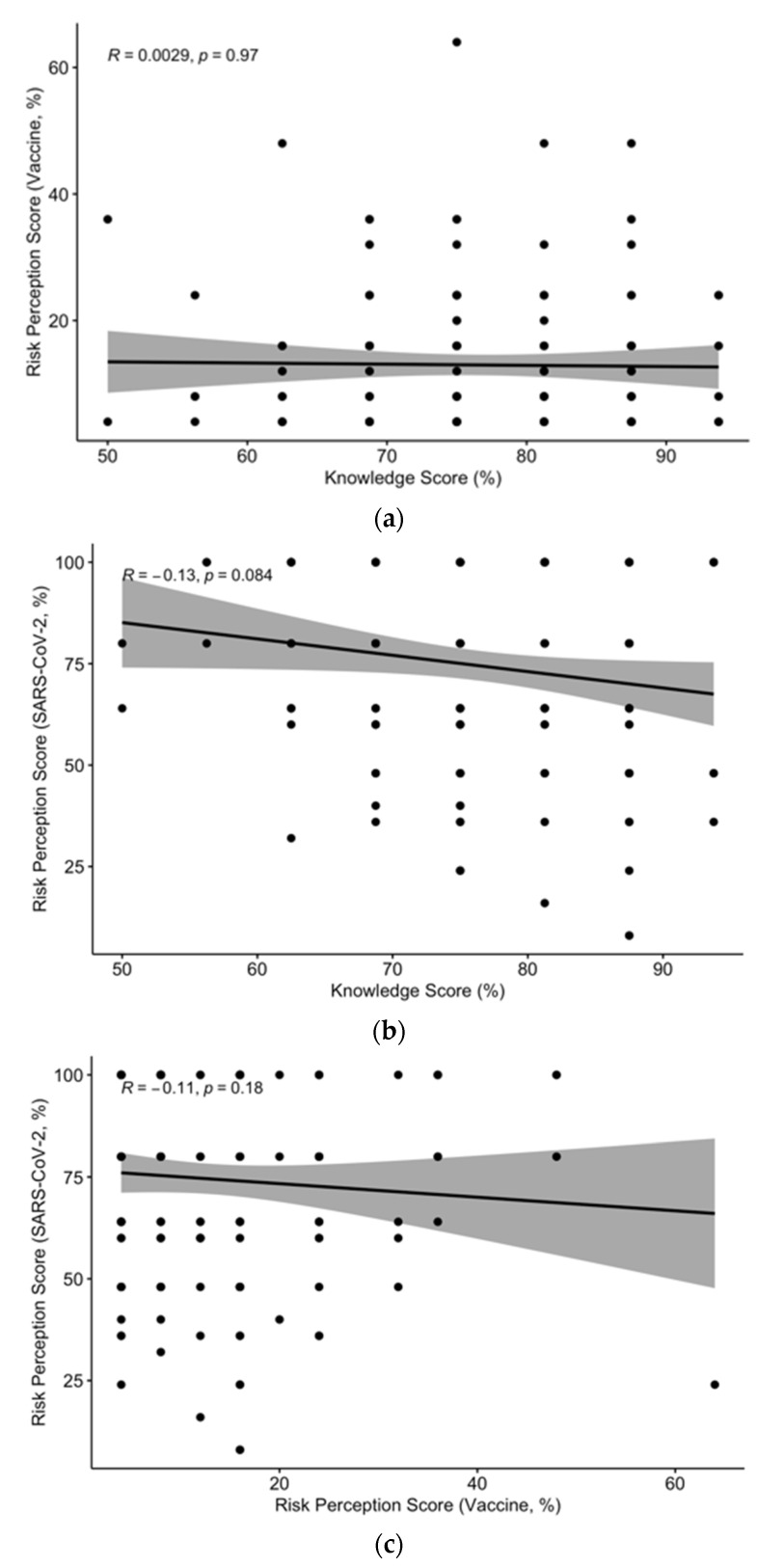
Scatter plots representing (left to right): (**a**) correlation between general knowledge score (%) and risk perception score (%) for SARS-CoV-2 vaccines (R = 0.0029, *p* = 0.97). (**b**) General knowledge score (%) and risk perception score for SARS-CoV-2 infection (R = −0.13, *p* = 0.084). (**c**) Risk perception score (%) for SARS-CoV-2 vaccines and for SARS-CoV-2 infection (R = −0.11, *p* = 0.18).

GKS was neither correlated with RPS for SARS-CoV-2 infection (R = −0.13, *p* = 0.084) nor for SARS-CoV-2 vaccines (R = 0.0029, *p* = 0.97). In other words, a better knowledge status (i.e., less misconceptions and/or less personal attitudes guiding the vaccine decisions) was not associated with a greater risk perception for SARS-CoV-2 infection and/or a lesser risk perception for the preventive measure represented by SARS-CoV-2 immunization (Figure 2).

Univariate analysis (Table 5) showed that a somewhat favorable attitude towards a mandatory status for SARS-CoV-2 vaccines in HCWs was significantly associated with a high-risk perception for COVID-19 among OPs (71.0% vs. 50.0%, *p* = 0.010), acknowledging COVID-19 as a diffuse (89.0% vs. 65.2%, *p* < 0.001) and severe disease (88.0% vs. 72.7%, *p* = 0.022), acceptance of payment/copayment for SARS-CoV-2 vaccine (81.0% vs. 50.0%, *p* = 0.007), reporting higher trust in vaccines (99.0% vs. 90.9%), common uptake of SIV (72.0% vs. 50.0%, *p* = 0.007), and assuming vaccine-hesitant HCWs as unfit for work, at least temporarily (83.0% vs. 47.0%, *p* < 0.001).

### 3.7. Multivariate Analysis

In regression analysis, acknowledging COVID-19 as a diffuse disorder (aOR 3.462, 95% CI 1.060 to 11.310), acceptance of payment/copayment (aOR 3.896, 95% CI 1.607 to 9.449), and recommending an unfit status for vaccine-hesitant workers (aOR 4.562, 95% CI 1.935 to 10.753) were identified as positive predictors for a proactive attitude towards recommending a mandatory SARS-CoV-2 vaccine status.

## 4. Discussion

A statutory mandate for SARS-CoV-2/COVID-19 vaccines has been advocated as an instrument to rapidly improve immunization rates, particularly among certain occupational groups [22,23], but it remains an ambiguous asset. Even though extensive vaccination mandates were instrumental and effective in improving vaccination rates for those in pediatric ages [24], it should be stressed that, before the start of the global vaccination campaigns, around 70% of the Western general population was somewhat favorable to get vaccinated as soon as possible [2,25,26]. In other words, under favorable conditions, a legal duty to get vaccinated to achieve herd immunity, might not be necessary [27]. On the other hand, a legal mandate to get vaccinated for COVID-19, coupled with coercive actions for individuals who opt-out of the vaccination program, might eventually impair the overall acceptance of vaccines, with obvious consequences [27]. However, the occurrence of the variants of concern (VoC), as well as uncertainties about the effective immunity elicited by some of the available formulates, may eventually increase the threshold for herd immunity, urging for more severe measures, particularly among high-risk groups. Not coincidentally, shortly after the accomplishment of this survey, the Italian government enabled a specific mandate for various occupational groups, particularly HCWs [28]. At the moment, the constitutional status of this decree remains unclear, and by 2 July 2021, several legal cases regarding vaccinations have been presented [29]. It should be stressed that, at the moment, Italian Law does not recognize a clear occupational mandate status for vaccinations other than that against tetanus—and only in specific high-risk groups (e.g., construction and agricultural workers, etc.), but vaccination rates remain largely inappropriate, with seroprevalence of protective tetanus antibodies well below 50% in individuals aged 50 years or older [30,31]. Even though the pre-pandemic legal framework would have urged OPs to judge unvaccinated workers as “unfit” for occupational settings, at high risk for biological agents, countered by a specific vaccine (e.g., HBV, pertussis, measles), this requirement remained largely unapplied [32,33,34]. For example, well before the SARS-CoV-2 pandemic, Di Martino et al. reported that among 347 enrolled HCWs, 57.3% reportedly missed diphtheritis–tetanus–pertussis vaccinations, while 50.1% had inappropriate statuses for measles–mumps–rubella (MMR) vaccinations, and 62.5% reportedly missed seasonal flu vaccinations [35]. As a consequence, some Italian regions have enforced specific decrees (e.g., Regional Decree of Emilia Romagna, no. 351/2018), and such interventions have seemly improved vaccination rates in targeted groups of HCWs [36]. However, such interventions did not represent a “formal” legal mandate, as the OPs were urged to consider unvaccinated HCWs as unfit for high-risk healthcare settings, leaving no space for more subjective (and permissive) interpretations.

On the contrary, at the moment, OPs are (and will be) professionally involved in the managing of COVID-19 vaccination mandates and its consequences; the appropriate assessments of their KAP may be of significant interest. In our study, nearly 90% of all participants exhibited some acceptance of SARS-CoV-2 vaccines. Moreover, 60.2% of them were somewhat favorable towards a mandatory status for SARS-CoV-2/COVID-19 vaccines. Interestingly, working as an OP for a main hospital (provincial or regional level) had no significant effect on the propensity towards a vaccination mandate. In other words, such attitude was seemly shared by all participants, irrespective of the healthcare setting they were more familiar with. These results may be explained by means of the shared experiences of the study participants, during the early stages of the pandemic, Italian nursing homes and long-term care facilities were severely hit, and OPs were regularly involved in managing the health and safety of HCWs [37,38,39].

Such estimates are hardly comparable with other KAP studies on SARS-CoV-2/COVID-19 vaccines [11,23,25,26,40,41,42,43,44], as we specifically inquired about a specific subset of medical professionals (i.e., OPs, particularly those having experience in healthcare settings), on the well-defined outcomes represented by their acceptance of the mandatory status. Interestingly, despite the ongoing debate on this topic [10,23,25,45], its actual acceptance by medical professionals has been scarcely investigated, particularly in Europe. Moreover, it is quite obvious that the support for mandatory vaccinations is both influenced by a baseline support and the specific timeframe of its assessment. For example, Giannouchos et al. [27] identified a large support base for the mandatory status among the general population (i.e., 74%), but it should be stressed that support for other mandatory vaccinations in the pre-pandemic period ranged from 65% to 97%.

The main effectors of such attitudes were identified, acknowledging the high occurrence of SARS-CoV-2 infections (aOR 3.462, 95% CI 1.060–11.310), better acceptance of the payment/copayment for SARS-CoV-2 vaccines (aOR 3.896, 95% CI 1.607–9.449), as well as a more rigorous attitude towards unvaccinated HCWs (aOR 4.562, 95% CI 1.935–10.753). On the one hand, such results are clearly consistent with the health belief model, i.e., an individual’s belief in a health threat, as well as belief in the effectiveness of the recommended health behavior or action are the main predictors for the likelihood that the person will adopt the behavior [46]. On the other hand, we failed to identify, as a predictive for acceptance of the mandatory status, a series of factors that previous studies strongly associated with the acceptance of SARS-CoV-2/COVID-19 vaccines, i.e., gender, history of personal uptake of SIV, knowledge status, previous interactions with the pathogen [47,48,49]. However, it should be stressed that the willingness to pay for a vaccine may be considered a summary index for perceived ease of access, awareness of the vaccine (including a trade-off of perceived pros and cons), but also the ability to pay for the vaccine [50]. In other words, such effectors may collectively reflect the struggle of sampled OPs to cope with a diffuse pathogen that is very difficult to contain, by means of non-pharmaceutical interventions, particularly in the workplaces, in a sampled population that was characterized by (a good understanding of) SARS-CoV-2 infection, and a very high acceptance of the vaccine. In such settings, vaccine mandates and coercive actions represented by a judgment of unfitness to work for vaccine-hesitant HCWs may be easily perceived as the easiest way to guarantee the safety of workers and patients [22,23,27,49,51].

Interestingly, the acceptance for SARS-CoV-2 vaccines was larger for mRNA formulates (i.e., 89.8%) than for vector-based (51.2%). This apparent lack of trust towards adenovirus-based formulates may be explained with the timing of our study. In fact, the survey was performed before the EMA granted emergency approval for ChAdOx1-S, but also before increased rates of venous thromboembolic events in recipients elicited more extensive contraindications, particularly among younger individuals [52,53]. Even though it is often believed that HCW attitudes towards vaccines cannot be negative because of their scientific backgrounds and medical training, these results were desirable, but not granted. Being that HCWs are at an increased risk of acquiring and transmitting the infection to susceptible and vulnerable patients in health and social care settings, they were among the first occupational groups to be vaccinated in most jurisdictions, particularly in the European Union [54,55,56]. Several previous studies have therefore observed their vaccine acceptance, and have identified a generally positive attitude towards SARS-CoV-2 vaccines. For example, in a large survey conducted among 1723 Italian healthcare workers, 67% (*n* = 1155) reported a willful acceptance of SARS-CoV-2/COVID-19 vaccination, 26% percent of the participants indicated some uncertainties, and 7% declared that they would refuse to be vaccinated [57]. Wide acceptance of SARS-CoV-2/COVID-19 vaccines was similarly reported by Szmyd et al. [58] (i.e., 94.4% among 252 Polish medical doctors), particularly by Verger et al. in a very large study involving 2768 HCWs from French speaking countries (i.e., France, Belgium, Canada): of them, 79.6% would either certainly or probably recommend a future COVID-19 vaccine to their patients; and 72.4% would certainly or probably agree to be vaccinated with it. Moreover, a subsequent study from Dzieciolowska et al. involving Canadian HCWs [47] confirmed a very high acceptance (80.9%), particularly when compared to other adult-administered vaccines [47]. On the other hand, numerous studies indicate that vaccine hesitancy exists among HCWs [48], and OPs are not exempt [12,13,32,34]. For example, in the study by Janssen et al., the willingness to get vaccinated against COVID-19 was 53.2%, but 19.8% and 14.1% were either not likely or not willing to get vaccinated at all [59], while a report by Gharpure et al. showed that a median of 37.5% of participating long-term care staff from the United States had accepted the first shot of a COVID-19 vaccine at the time of the study, demonstrating a low response to the vaccination campaign [60]. Moreover, even in the aforementioned study from Verger et al. a more accurate analysis of reported data identified some degree of vaccine hesitancy in 28.4% of respondents [47,48]. As HCWs represent a model for the general public, they may either be instrumental in overcoming vaccine hesitancy [61], or improperly spread false beliefs and misconceptions that may be eventually detrimental to global efforts to achieve high vaccination rates as soon as possible [12,13,32,34]. As highly diffusive new VoC of SARS-CoV-2 are rapidly raising the bar for the herd immunity threshold [62,63], vaccine hesitancy among HCWs, particularly OPs, may be a significant impediment in controlling the COVID-19 pandemic.

Despite the increasing amount of evidence, barriers to the COVID-19 vaccine uptake in HCWs are not completely understood, as they are quite inconsistent among the available studies. For example, in our study, the main perceived barriers were represented by perceptions of inappropriate vaccine safety (47.0%), followed by concerns on vaccine availability (24.0%), and actual vaccine efficacy (19.9%), with a large share of professionals exhibiting lack of confidence in the pharmaceutical industry (19.3%). Such estimates are quite consistent with available reports. For example, Di Gennaro et al. [57] identified doubts in vaccine efficacy and side effect fears (i.e., 76% and 85% of hesitant respondents) as main causes for the reluctance towards vaccination. Similarly, Spinewine et al. acknowledged potential side effects (60.9%), and the impression that the vaccine was developed too quickly (45.1%). Other reasons included the impression that the vaccine may not be effective against mutants, and the fact that respondents did not consider themselves at risk of serious complications from COVID-19 [64]. It should be stressed that, even though available SARS-CoV-2/COVID-19 vaccines represent the “end-stage” of a pipeline that began several years ago [65], as they were developed and delivered in less than a year, in emergency settings, concerns about their actual safety and effectiveness have been repeatedly associated with moderate acceptance, hesitancy, or reluctance [66,67], including in HCWs [47,68]. Interestingly, while safety and effectiveness concerns have been somewhat dismissed by available reports [69], worries on the availability of vaccines were properly addressed, as the “kick-off” of the vaccination administration was actually hampered by a short supply of vaccines [70], even during the summer of 2021, difficulties in maintaining appropriate deliveries by manufacturers have often been reported.

In regard to the perceived facilitators for SARS-CoV-2/COVID-19 vaccines, approximately two-thirds of participants acknowledged the will to protect friends and relatives (66.3%), followed by the perception of being at high risk for occupational infections (48.8%), with the aim to avoid the pathogen (47.0%), and a disease whose potential severity was perceived as particularly severe (43.4%). In other words, OPs (as well as other individuals) identified in the SARS-CoV-2 vaccines a potential “card to play” in order to protect themselves. Moreover, when we focus on facilitators associated with their occupational settings, the large majority of respondents identified vaccines as instrumental in coping with a series of significant issues that emerged during the first stage of the pandemic, at least in Italian healthcare settings [58,71,72,73,74,75], i.e., the limited reliability of most PPE (either because of their inadequate protection or limited availability), the limited usefulness of non-pharmaceutical interventions in healthcare settings, the inappropriate risk perceptions among many healthcare professionals. Moreover, OPs were aware that tracing and tracking (i.e., allowing early identification of secondary and subsequent cases) may be particularly complicated among HCWs, with obvious consequences in managing the pandemic, well beyond index occupational settings [75,76].

Such results were not unexpected, having been consistently reported by most available studies [25,26,44,47]. For example, Spinewine et al. [64] identified that the most important reasons why hesitant respondents would get vaccinated include the protection of loved ones and family (84.1%), colleagues (62.1%), or patients (60.1%), to get back to normal life (63.7%), to collectively get out of the crisis (60.6%), or to protect themselves (40.8%).

### Limitations of This Study

Despite this study’s novelty and its potential significance, it has several limits. Firstly, being an internet-based survey, it has implicit limits [77,78,79]. Conventional internet-based surveys—although reliable, cost-effective, and fast—are affected by an extensive “self-selection” of participants. As a consequence, certain sub-groups may be largely oversampled, impairing the overall reliability of collected results: subjects more accustomed to sharing personal information through internet access; individuals exhibiting proactive attitudes or greater knowledge about the assessed topic, etc. Similarly, not participating could be understood as a negative attitude or a lack of knowledge about the targeted topic [78]. In this regard, the potential self-selection of the participants may have been somewhat mitigated by targeting a very specific and therefore quite homogenous subgroup of medical professionals, i.e., OPs. Not coincidentally, while the majority of respondents were somewhat favorable toward enforcing legal mandates for SARS-CoV-2/COVID-19 vaccines among HCWs and high-risk groups, a considerable portion of the participants exhibited some degree of overconfidence in personal protective equipment, as valuable assets in preventing infections in healthcare settings. In other words, oversampling of medical professionals having favorable attitudes toward vaccination mandates could be ruled out.

Second, our sample was small, as it included a total of 166 OPs, i.e., 8.2% of the eligible population, but also 2.2% of all officially registered Italian OPs (*n* = 7825 by 3 July 2021), which could hardly be considered fully representative of the national level. On the other hand, according to the available statistics [80], a total of 1070 hospitals are, to date, affiliated with the Italian National Health Service, most of them assisted by one to three OPs. In other words, our sample included around 10% of the OPs working in these specific settings, including a far larger share of this subset of professionals. In fact, assuming, as a reference, the acceptance of SARS-CoV-2/COVID-19 vaccines as 67%, as reported by Di Gennaro et al. [57], an error of 5% (0.05), and a power of 95%, a minimum sample size equaling 1.96^2^ × 0.67 × (1−0.67)/0.05^2^ = 3.8416 × 0.67 × 0.33/0.0025 = 340 may be calculated. However, medical professionals participated from the whole country, with a greater representation of female subjects, those from Northern regions, and representation from various age groups was satisfyingly consistent with the reference population of Italian OPs [81].

Third, it is reasonable that some of the items assessed through the knowledge test may be affected by a significant social desirability bias, with participants not only reporting “common sense” answers, as previously discussed, but also those answers that may have been perceived as more “appropriate” to fit with the aim of the questionnaire. As such, potential bias was repeatedly identified in previous KAP studies, particularly among OPs [12,13,32,34,77], we cannot rule out that our results could have ultimately overstated the share of individuals i) having effective understanding of SARS-CoV-2 infections (and associated issues), and ii) actually acknowledging the vaccination mandate. In fact, this social desirability bias is indirectly suggested by some items on the knowledge test, particularly by the large share of respondents (around 44.0%) acknowledging adenovirus-based formulates as licensed by the EMA at the time of the survey, while the emergency authorization was still to come.

Fourth, even though discussion groups (e.g., by registering only subjects who received specific invitations by the managers, answering specific “selection” questions, etc.) involved in the recruitment of the study participants usually performed a preventive selection, we cannot rule out that some of the respondents did not fully adhere to our selection criteria, furtherly compromising the actual representativity of the sample.

## 5. Conclusions

In conclusion, OPs participating in our survey exhibited mostly favorable attitudes toward SARS-CoV-2 vaccines, and a large share of them endorsed vaccination mandates for HCWs. The primary explanatory variables for vaccination mandates were represented by acknowledging the extensive spreading of SARS-CoV-2 infection, a favorable attitude toward a payment/copayment for vaccination, and a decrease in the belief that unvaccinated subjects are fit to work in healthcare settings. In fact, sampled OPs had an extensive lack of trust in factors that may be reliable in settings other than healthcare, including non-pharmaceutical measures, extensive use of PPE, tracing and tracking of new cases, and potential exposures. As HCWs may be deliberately exposed to highly infectious patients even before a specific diagnosis of SARS-CoV-2, and appropriate PPE may fail to guarantee appropriate protection for several reasons (i.e., scarce quality, inappropriate protection, limited availability, etc.), vaccinations may be valuable instruments used to improve workplace safety, and vaccination mandates may quickly and more easily remove the barriers presented by vaccine hesitancy. On the other hand, our results suggest that OPs have a relatively good understanding of SARS-CoV-2/COVID-19-related issues. Their acceptance of available vaccine, particularly mRNA-based, may speed up global efforts to achieve high vaccination rates throughout workplaces.

## Figures and Tables

**Table 1:** General characteristics of 166 occupational physicians participating in the survey (January 2021).

Variable	No., %	Average ± SD
Gender		
Male	67 (40.4%)	
Female	99 (59.6%)	
Age (years)		49.1 ± 10.7
Age ≥ 50 years	60 (36.1%)	
Seniority (years)		22.2 ± 10.3
Seniority ≥ 15 years	134 (80.7%)	
Working as occupational physician in Hospital(s) affiliated with National Health Service	98 (59.0%)	
Geographical origin		
Northern Italy	60 (36.1%)	
Central Italy	52 (31.3%)	
Southern Italy	54 (32.5%)	
Any previous interaction with SARS-CoV-2		
Previous diagnosis in him/herself	17 (10.2%)	
Previous diagnosis in relatives	42 (25.3%)	
Information sources		
Conventional media (TV, journals, etc.)	36 (21.7%)	
New media (Wikis, social media, Twitter, etc.)	28 (16.9%)	
Websites from international and governmental agencies	146 (88.0%)	
Friend, relatives	8 (4.8%)	
Colleagues	3 (1.8%)	
Formation courses	117 (70.5%)	
Knowledge score (%)		76.3 ± 9.3
Higher knowledge score (>75.0%)	69 (41.6%)	

**Table 2:** Knowledge test: response distribution of the presented items proposed to the 166 occupational physicians participating in the survey and contributing to the assessment of the general knowledge score (GKS) (Cronbach’s Alpha = 0.717).

Statement	CORRECT ANSWER	No., %
More severe cases of COVID-19 occur in subjects ≥ 65 year-old and/or subjects affected by comorbidities	TRUE	149 (89.8%)
Main complications of COVID-19 are represented by respiratory distress syndrome	TRUE	155 (93.4%)
By January 2021, adenovirus-based vaccines were approved by the EMA	FALSE	73 (44.0%)
Present-day case-fatality-ratio of COVID-19 in Italy		
…is greater than 1 out of 10 affected cases (> 10%)	FALSE	8 (4.8%)
…accounts for 1 out of 10 affected cases (~10%)	FALSE	16 (10.8%)
…accounts for 1 out of 100 affected cases (~1%)	TRUE	66 (39.8%)
…accounts for 1 out of 1000 affected cases (~0.1%)	FALSE	45 (27.1%)
…remains unknown	FALSE	20 (12.0%)
mRNA-based SARS-CoV-2 vaccine ComiRNAty™ requires only one vaccination shot	FALSE	140 (84.3%)
Official efficiency of mRNA-based SARS-CoV-2 vaccine ComiRNAty™ is greater than 90%	TRUE	150 (90.4%)
Pleural ultrasonography is an efficient instrument in early diagnosis of SARS-CoV-2 interstitial pneumonia	TRUE	106 (63.9%)
SARS-CoV-2 is efficiently transmitted by cough	TRUE	161 (97.0%)
SARS-CoV-2 is mainly transmitted by contaminated blood	FALSE	154 (92.8%)
Hand washing reduces the risk for SARS-CoV-2 infections	TRUE	160 (96.4%)
All cases infected by SARS-CoV-2 develop COVID-19 symptoms	FALSE	162 (97.6%)
An efficient and etiologic treatment for COVID-19 has been made available	FALSE	147 (88.6%)
Latency of COVID-19 may reach 14 days	TRUE	147 (88.6%)
Gold standard for SARS-CoV-2 infection is represented by real-time quantitative polymerase chain reaction	TRUE	164 (98.8%)
Rapid antigen detection tests for SARS-CoV-2 infection are quite specific but scarcely sensitive	TRUE	113 (68.1%)
Temporarily, RNA-based SARS-CoV-2 vaccine ComiRNAty™ cannot be delivered to pregnant women	TRUE	94 (56.6%)

**Table 3:** Attitudes and practices of 166 OPs participating in the survey on COVID-19 vaccines (Italy, January 2021).

Variable	No., %	Average ± SD
Reported trust in vaccines (high/very high)	159 (95.8%)	
Reported acceptance of SIV (often/always)	105 (63.3%)	
Reported acceptance of COVID-19 vaccines		
mRNA vaccines	149 (89.8%)	
Adenovirus-based vaccines	85 (51.2%)	
any	150 (90.4%)	
How much would you pay for a SARS-CoV-2 vaccine?		
Nothing, vaccine should be provided for free	52 (31.3%)	
Participation to the expenditure	49 (29.5%)	
Up to EUR 10/dose	9 (5.4%)	
Between EUR 10 to 49/dose	9 (5.4%)	
Between EUR 50 to 99/dose	16 (9.6%)	
Between EUR 100 to 199/dose	16 (9.6%)	
EUR 200 or more/dose	15 (9.0%)	
Acceptance of SARS-CoV-2 immunization by means of…(agree/totally agree)		
…vaccines based on inactivated SARS-CoV-2	91 (54.8%)	
…adenovirus-based vaccines	85 (51.2%)	
…attenuated SARS-CoV-2	49 (29.5%)	
…vaccines based on SARS-CoV-2 mRNA	149 (89.8%)	
SARS-CoV-2 vaccine should be mandatory?		
No, I think that it is dangerous	1 (0.6%)	
No, it must be performed on a voluntary basis	36 (21.7%)	
No, it must be recommended to high-risk subjects	1 (0.6%)	
No, it must be recommended to high-risk subjects and HCWs	1 (0.6%)	
No, it must be recommended to high-risk subjects and high risk-workers, including HCWs	27 (16.3%)	
Yes, it should be made mandatory	55 (33.1%)	
Yes, it should be made mandatory with fines for hesitant	45 (27.1%)	
Occupational physicians should retain the vaccine-hesitant HCWs as…		
…still fit for work, as PPE are more efficient than vaccines in preventing SARS-CoV-2 infection	50 (30.1%)	
…unfit for work in high-risk settings, with temporary reassignment to low-risk tasks (if available)	83 (50.0%)	
…unfit for working in healthcare settings, permanently	33 (19.9%)	
**Risk perception**		
High risk for COVID-19 among occupational physicians	104 (62.7%)	
COVID-19 acknowledged as a common disease	132 (79.5%)	
COVID-19 acknowledged as a severe disease	136 (81.9%)	
mRNA vaccine side effects acknowledged as a frequently reported issue	7 (4.2%)	
mRNA vaccine side effects acknowledged as a severe issue	9 (5.4%)	
Risk perception score for COVID-19 (%)		74.5 ± 24.3
Risk perception score for mRNA vaccines (%)		13.0 ± 10.6

Note: PPE = personal protective equipment; HCW = healthcare workers.

**Table 4:** Perceived barriers and facilitators towards SARS-CoV-2 vaccination in occupational settings as reported by 166 occupational physicians participating in the survey (January 2021).

	**Total** **(No./166, %)**
Perceived barriers	
Inappropriate vaccine safety (perceived)	78 (47.0%)
Inappropriate vaccine efficacy (perceived)	33 (19.9%)
Inappropriate vaccine availability	40 (24.1%)
Lack of confidence in NHS	17 (10.2%)
Lack of confidence in NHS personnel	7 (4.2%)
Workers not acknowledging themselves among high-risk groups	20 (12.0%)
COVID-19 not acknowledged as a severe disease	30 (18.1%)
Higher confidence in alternative approach (i.e., hyperimmune plasma)	9 (5.4%)
Higher confidence in alternative approach (i.e., hydroxychloroquine)	8 (4.8%)
Lack of confidence in pharmaceutical industry	32 (19.3%)
Perceived facilitators	
Willingness to protect himself/herself	78 (47.0%)
Willingness to protect friends, relatives	110 (66.3%)
Willingness to avoid complications	57 (34.3%)
Willingness to avoid COVID-19	60 (36.1%)
Workers acknowledging themselves among high-risk groups	81 (48.8%)
COVID-19 acknowledged as a severe disease	72 (43.4%)
Lack of confidence in alternative treatments	4 (2.4%)
Lack of confidence in PPE	152 (91.6%)
Lack of confidence in HCW risk perception	142 (85.0%)
NPI are of limited reliability in healthcare settings	113 (68.1%)
Tracing and tracking of COVID-19 cases are unreliable in healthcare settings	100 (60.2%)

Note: NHS = National Health Service; NPI = non-pharmaceutical interventions.

**Table 5:** Association of individual characteristics with attitudes towards a mandatory status for SARS-CoV-2 vaccine in 166 Italian occupational physicians (January 2021). Adjusted odds ratios (aOR) were calculated with their respective 95% CI by means of a binary regression analysis model, including all factors that, in univariate analysis, were associated with a favorable attitude towards a mandatory status for SARS-CoV-2 vaccine with *p* < 0.05.

	Attitude Towards a Mandatory Status for SARS-CoV-2 Vaccines		Chi Squared Test *p* Value	aOR (95% CI)
	Somewhat Favorable (No./100, %)	Somewhat Not Favorable (No./66, %)		
Age ≥ 50 years	33 (33.0%)	27 (40.9%)	0.383	-
Seniority ≥ 15 years	80 (80.0%)	54 (81.8%)	0.929	-
Male sex	46 (46.0%)	21 (31.8%)	0.097	-
Being form Northern Italy	32 (32.0%)	28 (42.4%)	0.229	-
Working as occupational physician in hospital(s) affiliated with National Health Service	59 (59.6%)	39 (59.1%)	1.000	-
Previous diagnosis of COVID-19 in him/herself	10 (10.0%)	7 (10.6%)	1.000	-
Previous diagnosis of COVID-19 in relatives	25 (25.0%)	17 (25.8%)	1.000	-
Perceived high risk for COVID-19 among occupational physicians	71 (71.0%)	33 (50.0%)	0.010	2.332 (0.968; 5.617)
COVID-19 acknowledged as a common disease	89 (89.0%)	43 (65.2%)	< 0.001	3.462 (1.060; 11.310)
COVID-19 acknowledged as a severe disease	88 (88.0%)	48 (72.7%)	0.022	1.617 (0.463; 5.639)
mRNA vaccine side effects acknowledged as a frequently reported issue	3 (3.0%)	4 (6.1%)	0.572	-
mRNA vaccine side effects acknowledged as a severe issue	4 (4.0%)	5 (7.6%)	0.519	-
Acceptance of payment/copayment for SARS-CoV-2 vaccine	81 (81.0%)	33 (50.0%)	< 0.001	3.896 (1.607; 9.449)
Reported trust in vaccines (high/very high)	99 (99.0%)	60 (90.9%)	0.032	2.308 (0.167; 31.825)
Reported acceptance of SIV (often/always)	72 (72.0%)	33 (50.0%)	0.007	2.091 (0.926; 4.718)
Higher knowledge score	37 (37.0%)	32 (48.5%)	0.191	-
Information sources				
Conventional media (TV, journals, etc.)	24 (24.0%)	12 (18.2%)	0.485	-
New media (Wikis, social media, Twitter, etc.)	13 (13.0%)	15 (22.7%)	0.154	-
Websites from international and governmental agencies	87 (87.0%)	59 (89.4%)	0.826	-
Friend, relatives	7 (7.0%)	1 (1.5%)	0.213	-
Colleagues	2 (2.0%)	1 (1.5%)	1.000	-
Formation courses	73 (73.0%)	44 (66.7%)	0.483	-
Occupational physicians should retain the vaccine-hesitant HCWs as unfit for work	83 (83.0%)	31 (47.0%)	<0.001	4.562 (1.935; 10.753)

Note: HCW = healthcare workers; SIV = Seasonal Influenza Vaccine.

## Data Availability

The data presented in this study are available on request from the corresponding author.
